# Hypoxia Impairs CD8^+^ T Cell Fitness and Is Associated with a Dysfunctional CD8^+^ T Cell State in Pancreatic Cancer

**DOI:** 10.3390/cancers18101508

**Published:** 2026-05-08

**Authors:** Ashley M. Mello, Marina Pasca di Magliano, Kyoung Eun Lee

**Affiliations:** 1 Program in Immunology, University of Michigan, Ann Arbor, MI 48109, USA; 2 Rogel Cancer Center, University of Michigan, Ann Arbor, MI 48109, USA; 3 Department of Surgery, University of Michigan, Ann Arbor, MI 48109, USA; 4 Department of Cell and Developmental Biology, University of Michigan, Ann Arbor, MI 48109, USA; 5 Department of Pharmacology, University of Michigan, Ann Arbor, MI 48109, USA

**Keywords:** hypoxia, pancreatic cancer, CD8^+^ T cell, fibroblast, proliferation, apoptosis, differentiation, exhaustion

## Abstract

Pancreatic ductal adenocarcinoma (PDAC) is characterized by a dense stromal microenvironment and profound hypoxia, which contribute to therapeutic resistance. Using an in vitro system incorporating pancreatic cancer cells and cancer-associated fibroblasts (CAFs), we show that hypoxia suppresses CD8^+^ T cell accumulation and, in combination with cancer cell- and CAF-derived factors, further impairs T cell fitness by increasing cell death and reducing proliferation. Although the combination of hypoxia and cancer cell/CAF-derived factors enhances IFNγ and granzyme B expression in CD8^+^ T cells on a per-cell basis, the overall number of functional effector T cells is markedly reduced. Analysis of human PDAC single-cell RNA sequencing data corroborates these findings, revealing that CD8^+^ T cells enriched for hypoxia signatures exhibit elevated apoptosis and stress-response pathways. Furthermore, hypoxia is associated with downregulation of stemness-related genes and upregulation of terminal differentiation markers. Together, these data suggest that the integration of intrinsic hypoxic responses and extrinsic cues from tumor cells and CAFs impairs CD8^+^ T cell fitness and correlates with a terminally differentiated, dysfunctional T cell state.

## Simple Summary

Pancreatic cancer is a highly lethal disease characterized by a dense, stroma-rich, and hypoxic tumor microenvironment. Here, we show that hypoxia, together with pancreatic cancer cell- and fibroblast-derived factors, limits CD8^+^ T cell accumulation by suppressing proliferation and promoting cell death. Although hypoxia can enhance IFNγ and granzyme B expression on a per-cell basis, the overall pool of functional effector T cells is reduced. Analysis of human pancreatic cancer datasets supports these findings, revealing that CD8^+^ T cells enriched for hypoxia signatures exhibit increased apoptosis and stress-response pathways, alongside diminished stem-like properties and features of terminal differentiation. Collectively, these results demonstrate that hypoxia and tumor- and fibroblast-derived signals cooperate to impair T cell fitness in pancreatic cancer and may inform strategies to improve immunotherapy.

## 1. Introduction

Pancreatic ductal adenocarcinoma (PDAC) is a lethal disease that is refractory to T cell-directed immunotherapies [1,2,3]. A defining feature of PDAC is its extensive desmoplastic stroma, composed of fibroblasts, extracellular matrix (ECM), and immune cells [4]. Cancer-associated fibroblasts (CAFs) represent a stromal component and play a central role in establishing an immunosuppressive tumor microenvironment (TME) and promoting disease progression [5]. CAFs exert these effects through the secretion of growth factors, cytokines, and chemokines, as well as through the production and remodeling of the ECM [6,7].

Solid tumors frequently experience hypoxia, or oxygen deprivation, due to abnormal vasculature and high oxygen/nutrient demand [8,9]. Tumor hypoxia is associated with cancer progression and therapy resistance, inducing adaptive responses in both malignant and stromal cells [10,11]. PDAC is among the most hypovascular and hypoxic tumor types [12,13,14]; however, the mechanisms by which hypoxia regulates stromal cell function and tumor–stroma interactions in PDAC remain incompletely understood.

T cells are critical mediators of anti-tumor immunity, yet, in PDAC, they are largely dysfunctional or exhausted [2,15,16]. While hypoxia has been shown to directly impact CD8^+^ T cell proliferation and function, these intrinsic responses vary depending on the timing and duration of exposure [17,18,19,20]. Adding complexity, T cell responses are also modulated by extrinsic signaling from the surrounding TME [2,21,22]. Hypoxia alters the phenotype and function of tumor cells and CAFs, thereby modifying the extrinsic signaling from these cells [6,23,24,25,26,27]. The net T cell response in a hypoxic TME is, thus, a result of both intrinsic responses and extrinsic regulation by surrounding cells. In the context of PDAC, the impact of hypoxia on T cell accumulation and function remains largely unknown.

In this study, we show that hypoxia intrinsically reduces CD8^+^ T cell proliferation, decreases IFNγ production, and increases the expression of co-inhibitory receptors. We further demonstrate that secreted factors from pancreatic cancer cells and CAFs cooperate with hypoxia, resulting in increased T cell death, decreased proliferation, and increased expression of effector molecules and co-inhibitory receptors. Corroborating these in vitro findings, analysis of a human PDAC single-cell RNA sequencing (scRNA-seq) dataset reveals that CD8^+^ T cells enriched for hypoxia signatures exhibit elevated apoptosis and stress-response programs, downregulation of stemness markers, and upregulation of terminal exhaustion-related genes. Our findings demonstrate that hypoxia and tumor–CAF signals together limit CD8^+^ T cell accumulation and enforce CD8^+^ T cell dysfunction in PDAC.

## 2. Materials and Methods

### 2.1. Mice

All animal studies were conducted in accordance with protocols approved by the Institutional Animal Care and Use Committee at the University of Michigan (PRO00012213). Wild-type C57BL/6 mice (stock #000664, Jackson Laboratory, Bar Harbor, ME, USA), both male and female, were used as a source of splenic CD8^+^ T cells between 8 and 12 weeks of age.

### 2.2. Cell Lines and Cell Culture

The murine pancreatic cancer cell line 7940B (provided by Dr. Gregory L. Beatty) [28] was derived from primary KPC (*Kras^LSL-G12D/+^*; *Trp53^LSL-R172H/+^*; *Pdx1-Cre*) PDAC. The FB1 CAF line was established from a *p48-Cre*; *TetO-Kras^G12D^*; *Rosa26^LSL-rtTa-EGFP/+^*; *Trp53^LSL-R172H/+^* mouse [29] via fluorescence-activated cell sorting of PDGFRα^+^; EPCAM^−^; EGFP^−^ cells [23,26,27,30]. Both cell lines were maintained in DMEM (#MT10017CV, Corning, Glendale, AZ, USA) supplemented with 10% heat-inactivated fetal bovine serum (#A5209501, Gibco, Waltham, MA, USA) and 1% penicillin-streptomycin (#15140122, Gibco), and experiments were performed using cells within 15–20 passages. Routine mycoplasma testing was carried out using the MycoAlert PLUS Mycoplasma Detection Kit (#LT07-710, Lonza, Basel, Switzerland).

### 2.3. Conditioned Media (CM) Generation

For monocultures, 1.5 × 10^5^ 7940B or FB1 cells were seeded per 6-well plate (#657160, Greiner Bio-One, Monroe, NC, USA). For co-cultures, 7.5 × 10^4^ of each cell type were plated together. Cells were cultured under either 21% or 1% O_2_ for 48 h in T cell medium, consisting of RPMI (#10-040-CV, Corning) supplemented with 10% heat-inactivated FBS (#A5209501, Gibco), 1% penicillin-streptomycin (#15140122, Gibco), 1% L-glutamine (#25-030-081, Gibco), 1% MEM non-essential amino acids (#11-140-050, Gibco), 1 mM sodium pyruvate (#11-360-070, Gibco), 10 mM HEPES solution (#H0887, Sigma-Aldrich, St. Louis, MO, USA), and 50 µM 2-mercaptoethanol (#21-985-023, Gibco). CM was collected and centrifuged at 1000× *g* for 10 min at 4 °C, and the supernatants were harvested.

### 2.4. CD8^+^ T Cell Isolation and Activation

CD8^+^ T cells were isolated from the spleens of C57BL/6 mice (#000664, Jackson Laboratory) using the MojoSort Mouse CD8 T Cell Isolation Kit (#480035, BioLegend, San Diego, CA, USA). Cells were activated for 24 h using plate-bound anti-CD3 (145-2C11, 5 μg/mL, #100359, BioLegend) and anti-CD28 (37.51, 5 μg/mL, #102121, BioLegend) in T cell medium supplemented with IL-2 (10 ng/mL, #575402, BioLegend). After activation, the cells were transferred to either fresh medium or a 1:1 mixture of fresh medium and CM in 96-well round-bottom plates (#351177, Falcon, Glendale, AZ, USA) with IL-2 (10 ng/mL) under either 21% or 1% O_2_. After 24 h, the cells were passaged and maintained under the same conditions for an additional 48 h (72-h exposure in total).

### 2.5. Flow Cytometry

The cells were washed with FACS buffer (PBS containing 0.5% FBS and 0.2 mM EDTA). Fc receptors were blocked using anti-mouse CD16/CD32 antibodies (#156604, BioLegend) for 5 min on ice. Surface staining was performed using fluorophore-conjugated antibodies for 30 min on ice. Dead cells were excluded by staining with a fixable viability dye (#423102, BioLegend) for 15 min at room temperature. Intracellular staining was carried out using the True-Nuclear Transcription Factor Buffer Set (#424401, BioLegend) following the manufacturer’s protocol.

For the cytokine measurements, cells were stimulated for 4 h under their respective oxygen conditions with PMA (5 ng/mL, #10008014, Cayman Chemical, Ann Arbor, MI, USA), ionomycin (0.5 μg/mL, #11932, Cayman Chemical), and Brefeldin A (10 μg/mL, #11861, Cayman Chemical). For the proliferation assays, naive CD8^+^ T cells were loaded with 5 µM carboxyfluorescein succinimidyl ester (CFSE) (#13-0850-U500, Cytek Biosciences, Fremont, CA, USA) for 15 min at room temperature and quenched according to the manufacturer’s instructions. For apoptosis detection, cells were washed with Annexin V Binding Buffer (#422201, BioLegend) and incubated with FITC Annexin V and 7AAD (#640922, BioLegend) for 15 min at room temperature in the dark and analyzed within 1 h.

Data were acquired on a ZE5 Cell Analyzer (Bio-Rad, Hercules, CA, USA), and the analyses were conducted using FlowJo 10.10.0. The antibodies used for flow cytometry were as follows: AF700-conjugated anti-CD8a (53-6.7, 1:300, #100730, BioLegend), PE-conjugated anti-Ki67 (16A8, 1:200, #652403, BioLegend), APC-conjugated anti-PD-1 (29F.1A12, 1:200, #135209, BioLegend), PerCP-Cy5.5-conjugated anti-TIM-3 (RMT3-23, 1:100, #119717, BioLegend), FITC-conjugated anti-LAG-3 (C9B7W, 1:100, #11-2231-82, Thermo Fisher Scientific, Waltham, MA, USA), APC-conjugated anti-IFNγ (XMG1.2, 1:100, #505809, BioLegend), PerCP-Cy5.5-conjugated anti-TNFα (MP6-XT22, 1:100, #506321, BioLegend), and PE-Cy7-conjugated anti-granzyme B (NGZB, 1:200, #25-8998-82, Thermo Fisher Scientific).

### 2.6. Single-Cell RNA Sequencing Analysis

A previously published scRNA-seq dataset comprising 16 human PDAC tumors [31] was analyzed (NIH dbGaP accession: PHS002071.v1.p1). Downstream analyses were conducted in R (v4.5.0) using RStudio (v2024.12.1+ 563) and Seurat (v5.2.1). Hypoxia scores were calculated using the MSigDB “HALLMARK_HYPOXIA” gene set via Seurat’s AddModuleScore function. CD8^+^ T cells were stratified into quartiles based on the hypoxia scores, with the top quartile designated as hypoxic and the bottom quartile as normoxic. Differential gene expression analysis was performed using FindMarkers with the Mann–Whitney test and Bonferroni correction. Gene set enrichment analysis was carried out using fgsea (v1.34.0), with Hallmark gene sets obtained from MSigDB (v7.5.1). Enrichment was computed using the fgseaMultilevel function with the adaptive multilevel splitting algorithm and Benjamini–Hochberg correction.

### 2.7. Statistical Analysis

Statistical analyses were performed using GraphPad Prism 10. Data normality was assessed using the D’Agostino–Pearson and/or Shapiro–Wilk tests. Depending on the data distribution and experimental design, Mann–Whitney tests or two-way ANOVA were applied, as indicated in the figure legends. The Holm–Sidak correction was used for multiple comparisons. A *p*-value < 0.05 was considered statistically significant. The sample sizes were not predetermined by statistical methods, the experiments were not randomized, and the investigators were not blinded during the data collection or analysis.

## 3. Results

### 3.1. Hypoxia and Cancer Cell/CAF-Derived Factors Reduce CD8^+^ T Cell Proliferation and Survival

**Figure 1:**
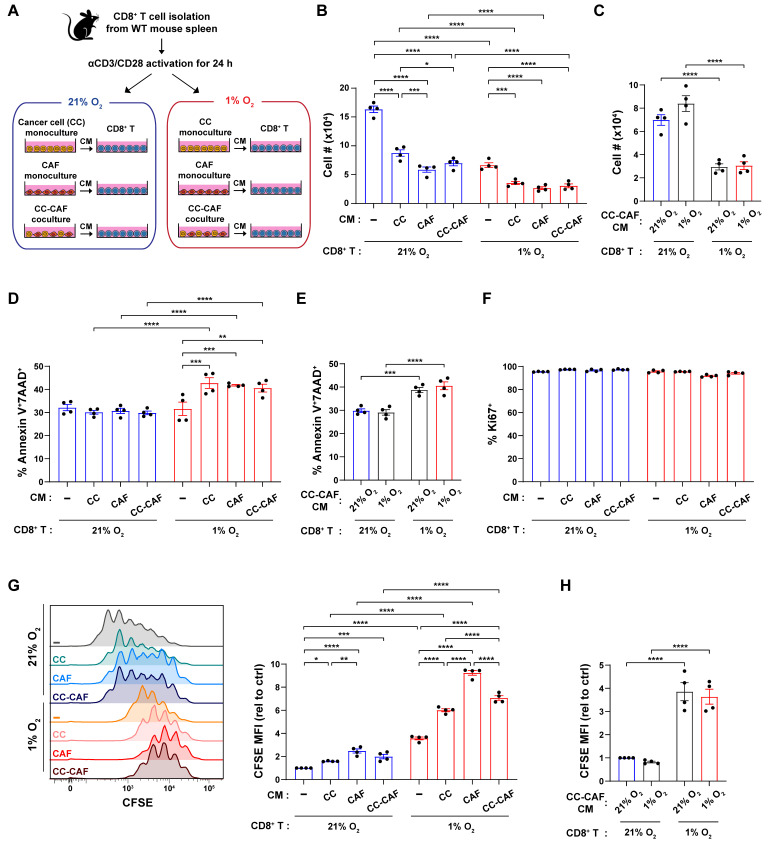
Hypoxia and cancer cell/CAF-derived factors reduce CD8^+^ T cell proliferation and survival. (**A**) Experimental workflow. Conditioned media (CM) was generated from mouse pancreatic cancer cells (CCs) and CAFs cultured independently (monoculture) or in co-culture under normoxia (21% O_2_) or hypoxia (1% O_2_) for 48 h. Splenic CD8^+^ T cells from wild-type (WT) mice were activated with anti-CD3/CD28 and IL-2. After 24 h, T cells were transitioned into either fresh medium (control) or a 1:1 mixture of fresh medium and CM and maintained under 21% or 1% O_2_. T cells were analyzed by flow cytometry 72 h post-treatment. (**B**,**C**) Absolute number of live CD8^+^ T cells. (**D**,**E**) Percentage of Annexin V^+^7AAD^+^ cells among total CD8^+^ T cells. (**F**) Percentage of Ki67^+^ cells among live CD8^+^ T cells. (**G**,**H**) Representative histograms and quantification of CFSE dilution in live CD8^+^ T cells. MFI, median fluorescence intensity. Each dot represents an independent experiment. Data are mean ± SEM. *p*-values were determined by two-way ANOVA with Holm–Sidak post hoc. * *p* < 0.05; ** *p* < 0.01; *** *p* < 0.001; **** *p* < 0.0001.

To investigate the impact of hypoxia on CD8^+^ T cell expansion and function, we sought to distinguish between intrinsic hypoxic responses and extrinsic regulation by neighboring cells, including tumor cells and CAFs. We established an in vitro system using conditioned media (CM) harvested from pancreatic cancer cell (CC) monocultures, CAF monocultures, or CC–CAF co-cultures, each maintained under either normoxia (21% O_2_) or hypoxia (1% O_2_). CD8^+^ T cells were isolated from wild-type mouse spleens and activated with anti-CD3 and anti-CD28 in the presence of IL-2 for 24 h. Following activation, T cells were passaged and cultured for an additional 72 h in the respective CM or fresh media control under oxygen-matched conditions, pairing normoxic CM with 21% O_2_ T cell culture and hypoxic CM with 1% O_2_ T cell culture (Figure 1A).

Under normoxic conditions, CM derived from CC and CAF monocultures, as well as CC–CAF co-cultures, significantly reduced the total number of live CD8^+^ T cells compared with the fresh media control (Figure 1B). Hypoxia alone inhibited CD8^+^ T cell accumulation, as T cells cultured in fresh media under hypoxia exhibited substantially lower cell numbers than those maintained under normoxia (Figure 1B). This suppressive effect was further exacerbated when CD8^+^ T cells were cultured in CM under hypoxic conditions, resulting in significantly fewer cells across all CM conditions compared with the hypoxic fresh media control (Figure 1B). To determine whether normoxic versus hypoxic CC–CAF co-cultures differentially regulate CD8^+^ T cell expansion, we treated CD8^+^ T cells with CM generated under normoxic or hypoxic conditions and cultured them under both oxygen conditions. CM from normoxic and hypoxic co-cultures exerted comparable effects on CD8^+^ T cell expansion under both normoxic and hypoxic conditions (Figure 1C). These data indicate that hypoxia and tumor- and CAF-derived soluble factors independently inhibit CD8^+^ T cell expansion and that their combined effects further impair CD8^+^ T cell accumulation.

To determine the mechanisms underlying this impaired expansion, we assessed CD8^+^ T cell death and proliferation. Annexin V/7AAD staining revealed a significant increase in cell death when T cells were exposed to the combination of CM and hypoxia; notably, neither CM nor hypoxia alone was sufficient to significantly alter cell death compared with the normoxic fresh media control (Figure 1D). No difference in cell death was observed between CM from normoxic and hypoxic CC–CAF co-cultures (Figure 1E). Furthermore, over 90% of CD8^+^ T cells across all experimental conditions remained positive for the proliferation marker Ki67, implicating minimal impact of hypoxia on senescence in this context (Figure 1F).

Analysis of CFSE dilution demonstrated that, under normoxic conditions, CM from CCs and CAFs suppressed CD8^+^ T cell division. Hypoxia alone also reduced T cell division, even in fresh media. Furthermore, CM exposure under hypoxic conditions led to an additional reduction in CD8^+^ T cell division compared with hypoxia alone (Figure 1G). Proliferation was similarly reduced by CM from normoxic and hypoxic CC–CAF co-cultures (Figure 1H). Collectively, these findings demonstrate that hypoxia and cancer cell/CAF-derived factors cooperatively impair CD8^+^ T cell expansion by suppressing proliferation and promoting cell death.

### 3.2. Hypoxia, Cancer Cells, and CAFs Differentially Regulate Effector Function of CD8^+^ T Cells

**Figure 2:**
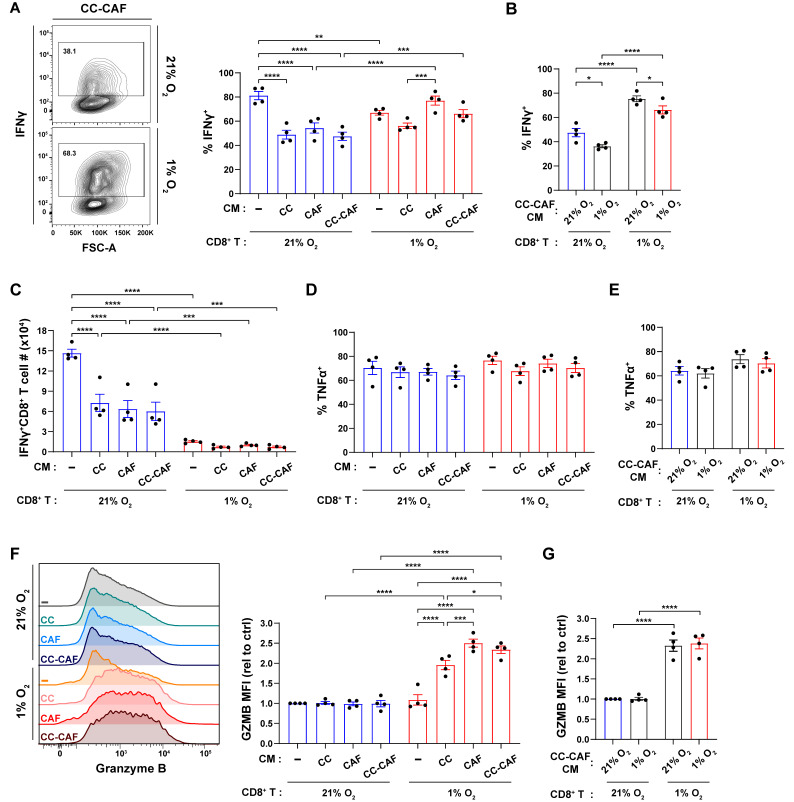
Hypoxia, cancer cells, and CAFs differentially regulate effector function of CD8^+^ T cells. CD8^+^ T cells were activated and cultured, as shown in Figure 1A, restimulated with PMA/ionomycin for 4 h, and analyzed by flow cytometry. (**A**,**B**) Representative flow plots and percentage of IFNγ^+^ cells among live CD8^+^ T cells. (**C**) Absolute number of live IFNγ^+^CD8^+^ cells. (**D**,**E**) Percentage of TNFα^+^ cells among live CD8^+^ T cells. (**F**,**G**) Representative histograms and quantification of granzyme B (GZMB) MFI in live CD8^+^ T cells. Each dot represents an independent experiment. Data are mean ± SEM. *p*-values were determined by two-way ANOVA with Holm–Sidak post hoc. * *p* < 0.05; ** *p* < 0.01; *** *p* < 0.001; **** *p* < 0.0001.

To assess the effects of hypoxia and tumor/CAF-derived factors on CD8^+^ T cell effector function, CD8^+^ T cells were activated and cultured, as described in Figure 1A, restimulated with PMA and ionomycin, and analyzed for effector molecule expression. Hypoxia alone significantly reduced IFNγ expression in CD8^+^ T cells cultured in fresh media compared with normoxia (Figure 2A). Under normoxic conditions, CM from CCs, CAFs, or CC–CAF co-cultures suppressed IFNγ expression (Figure 2A). In contrast, under hypoxic T cell culture conditions, CM did not further reduce IFNγ expression relative to fresh media (Figure 2A).

Notably, in the presence of CM from CAF monocultures or CC–CAF co-cultures, the hypoxic T cell culture resulted in higher IFNγ expression compared with the corresponding normoxic conditions (Figure 2A). To determine whether hypoxia alters the composition of factors released by tumor cells and CAFs to differentially modulate T cell effector function, we compared CM generated from CC–CAF co-cultures under normoxic versus hypoxic conditions. CD8^+^ T cells were treated with CM from either condition and cultured under both normoxic and hypoxic environments. Importantly, CM derived from hypoxic CC–CAF co-cultures resulted in a significant reduction in IFNγ^+^ T cell frequencies compared with CM generated under normoxia, regardless of the oxygen conditions of the recipient T cells (Figure 2B). These findings indicate that tumor cells and CAFs produce factors that suppress IFNγ expression and that hypoxic exposure of these cells further enhances this suppressive capacity. However, CD8^+^ T cell exposure to hypoxia partially attenuated this suppression, resulting in a higher frequency of IFNγ^+^ cells relative to normoxic T cell cultures treated with CM.

Of note, despite this relative increase in IFNγ^+^ cell frequency, the absolute number of IFNγ^+^ CD8^+^ T cells was substantially lower in all hypoxic conditions compared with their normoxic counterparts (Figure 2C and Figure S1), likely due to impaired T cell proliferation and survival (Figure 1B–H). Thus, hypoxia and tumor/CAF-derived factors collectively diminish the overall pool of functional effector CD8^+^ T cells.

In contrast to IFNγ, TNFα expression was unaffected by either hypoxia or CM (Figure 2D,E). However, granzyme B (GZMB) expression showed a distinct pattern: while hypoxia alone did not change GZMB levels in fresh media, and CM had no effect under normoxia, the combination of hypoxia and any CM significantly increased GZMB expression (Figure 2F). There was no significant difference between the effects of normoxic versus hypoxic CM on GZMB levels (Figure 2G), suggesting that hypoxic exposure of tumor cells and CAFs does not further enhance their capacity to induce GZMB expression. Collectively, these results demonstrate that hypoxia and tumor/CAF-derived factors cooperate to enhance GZMB expression, while hypoxia-driven changes in the tumor–CAF secretome selectively impact IFNγ regulation.

### 3.3. Hypoxia and Tumor–CAF Signals Cooperatively Upregulate Co-Inhibitory Receptors on CD8^+^ T Cells

**Figure 3:**
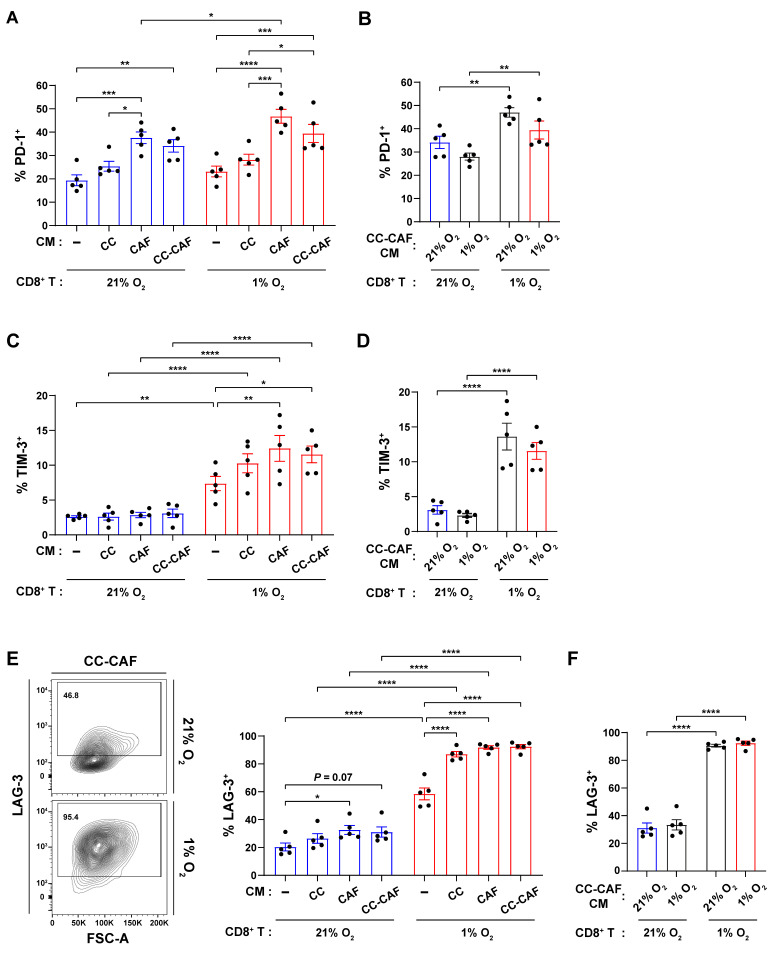
Hypoxia and tumor–CAF signals cooperatively upregulate co-inhibitory receptors on CD8^+^ T cells. CD8^+^ T cells were activated and cultured, as shown in Figure 1A, and analyzed by flow cytometry. (**A**,**B**) Percentage of PD-1^+^ cells among live CD8^+^ T cells. (**C**,**D**) Percentage of TIM-3^+^ cells among live CD8^+^ T cells. (**E**,**F**) Representative flow plots and percentage of LAG-3^+^ cells among live CD8^+^ T cells. Each dot represents an independent experiment. Data are mean ± SEM. *p*-values were determined by two-way ANOVA with Holm–Sidak post hoc. * *p* < 0.05; ** *p* < 0.01; *** *p* < 0.001; **** *p* < 0.0001.

We next investigated the impact of hypoxia and tumor/CAF-derived factors on the expression of co-inhibitory receptors on CD8^+^ T cells. We observed that hypoxia alone did not alter PD-1 expression (Figure 3A). However, CM from CAF monocultures or CC–CAF co-cultures increased PD-1 expression under both normoxic and hypoxic CD8^+^ T cell culture conditions (Figure 3A). Notably, CD8^+^ T cells treated with CAF-derived CM and cultured under hypoxia exhibited significantly higher PD-1 levels compared with their normoxic counterparts (Figure 3A). PD-1 levels were similar in CD8^+^ T cells treated with CM generated under normoxic or hypoxic conditions (Figure 3B), indicating that hypoxic exposure of tumor cells and CAFs does not further influence their capacity to promote PD-1 expression. Together, these results suggest that T cell-intrinsic hypoxic responses and CAF-derived signals contribute to the upregulation of PD-1.

We observed a distinct regulatory pattern for TIM-3. Unlike PD-1, TIM-3 expression was induced by hypoxia alone in CD8^+^ T cells cultured in fresh media. CAF or CC–CAF CM further potentiated TIM-3 upregulation when CD8^+^ T cells were cultured under hypoxia but had no effect under normoxia (Figure 3C). The oxygen conditions under which the CM was generated did not significantly influence TIM-3 expression (Figure 3D). These findings indicate that hypoxia and CAF-derived signals cooperatively enhance TIM-3 expression.

LAG-3 expression was similarly induced by hypoxia alone (Figure 3E). However, distinct from the TIM-3 phenotype, CAF and CC–CAF CM increased LAG-3 expression even in normoxic T cell cultures (Figure 3E). When T cells were subjected to hypoxia, the addition of CC, CAF, or CC–CAF CM all further significantly increased LAG-3 expression (Figure 3E). The oxygen conditions of CM did not affect LAG-3 expression (Figure 3F). Collectively, these data demonstrate that hypoxia and tumor cell/CAF-derived factors cooperatively promote co-inhibitory receptor expression on CD8^+^ T cells.

### 3.4. Hypoxic CD8^+^ T Cells Exhibit Elevated Apoptosis and Stress Signatures in PDAC

**Figure 4:**
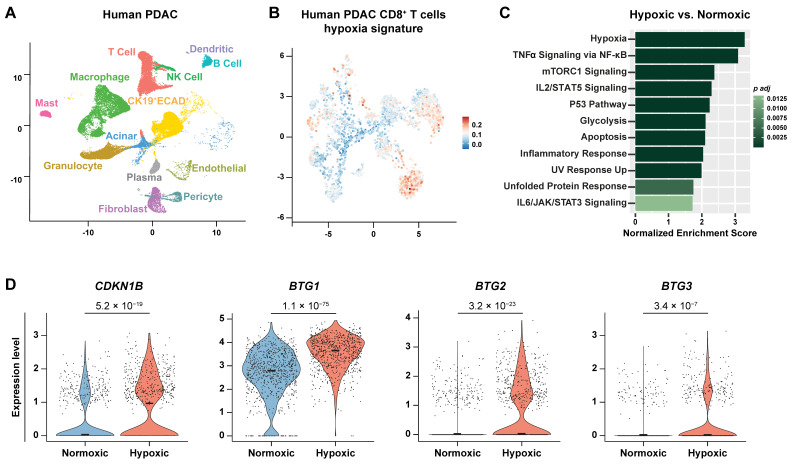
Hypoxic CD8^+^ T cells exhibit elevated apoptosis and stress signatures in PDAC. Analysis of the human PDAC scRNA-seq dataset (*n* = 16). (**A**) Uniform manifold approximation and projection (UMAP) showing major cell populations. The CK19^+^ (Cytokeratin19) ECAD^+^ (Ecadherin) cluster represents ductal/ductal-like malignant cells. (**B**) UMAP of CD8^+^ T cells colored by a module score for the MSigDB Hallmark hypoxia signature (red, highest; blue, lowest). CD8^+^ T cells were classified as hypoxic (top quartile) or normoxic (bottom quartile) based on the hypoxia module score. (**C**) Waterfall plot of Hallmark pathways enriched in hypoxic versus normoxic CD8^+^ T cell clusters. No pathways were enriched in the normoxic cluster. *p adj* = Benjamini–Hochberg-adjusted *p*-value. (**D**) Violin plots of *CDKN1B*, *BTG1*, *BTG2*, and *BTG3* expression in the normoxic and hypoxic CD8^+^ T cell clusters. *p*-values were determined by the Mann–Whitney test with Bonferroni correction.

PDAC is characterized by profound hypoxia; however, substantial intratumoral heterogeneity in oxygenation exists [23,32]. To characterize CD8^+^ T cells residing in hypoxic tumor regions in vivo and compare them with those in normoxic regions, we analyzed a scRNA-seq dataset comprising 16 human PDAC tumors [31] (Figure 4A). We stratified CD8^+^ T cells based on hypoxia exposure by mapping the Hallmark hypoxia gene set (MSigDB; 200 genes) [33] onto the dataset using gene module scoring (Figure 4B). Cells were classified as hypoxic if their module score fell within the top quartile and as normoxic if within the bottom quartile.

Consistent with our in vitro observation of increased cell death under hypoxia (Figure 1D), the “APOPTOSIS” and “P53 PATHWAY” signatures were significantly enriched in the hypoxic CD8^+^ T cell cluster (Figure 4C). In addition to the “HYPOXIA” signature, other stress-response pathways, including “UNFOLDED PROTEIN RESPONSE” and “UV RESPONSE UP”, were also enriched in hypoxic CD8^+^ T cells (Figure 4C). Furthermore, the hypoxic cluster exhibited enrichment for “TNFA SIGNALING VIA NFKB”, “IL2 STAT5 SIGNALING”, “INFLAMMATORY RESPONSE”, and “IL6 JAK STAT3 SIGNALING”, reinforcing the link between hypoxia and inflammation (Figure 4C).

In line with the reduced cell division under hypoxia observed in vitro (Figure 1G), hypoxic CD8^+^ T cells expressed significantly higher levels of *CDKN1B* (encoding p27), a cell-cycle inhibitor, as well as *BTG1*, *BTG2*, and *BTG3*, which are negative regulators of proliferation (Figure 4D). Together, these findings indicate that hypoxia is associated with suppressed proliferation and enhanced cell death in CD8^+^ T cells in PDAC.

### 3.5. Hypoxic CD8^+^ T Cells Correlate with Reduced Stemness and Enhanced Terminal Differentiation in PDAC

**Figure 5:**
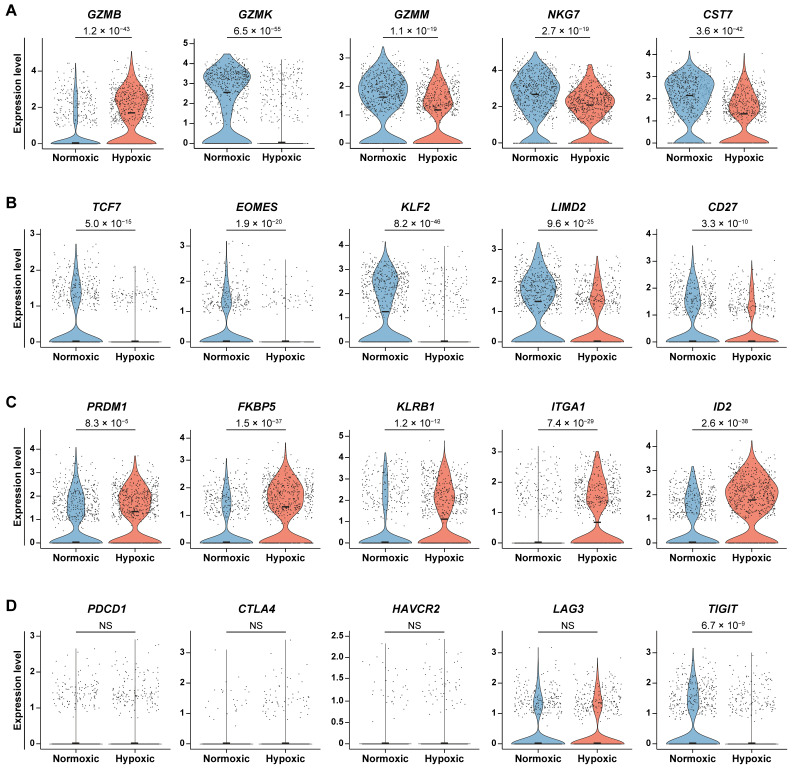
Hypoxic CD8^+^ T cells correlate with reduced stemness and enhanced terminal differentiation in PDAC. Analysis of CD8^+^ T cells from the human PDAC scRNA-seq dataset (*n* = 16). (**A**) Violin plots of cytotoxicity-related genes (*GZMB*, *GZMK*, *GZMM*, *NKG7*, and *CST7*) in the normoxic and hypoxic CD8^+^ T cell clusters. (**B**) Violin plots of progenitor-associated genes (*TCF7*, *EOMES*, *KLF2*, *LIMD2*, and *CD27*) in the normoxic and hypoxic CD8^+^ T cell clusters. (**C**) Violin plots of terminal differentiation-associated genes (*PRDM1*, *FKBP5*, *KLRB1*, *ITGA1*, and *ID2*) in the normoxic and hypoxic CD8^+^ T cell clusters. (**D**) Violin plots of co-inhibitory receptor genes (*PDCD1*, *CTLA4*, *HAVCR2*, *LAG3*, and *TIGIT*) in the normoxic and hypoxic CD8^+^ T cell clusters. *p*-values were determined by the Mann–Whitney test with Bonferroni correction. NS, not significant.

We next examined the functional and exhaustion states of normoxic and hypoxic CD8^+^ T cells in the human PDAC scRNA-seq dataset. Consistent with our in vitro observation (Figure 2F), *GZMB* expression was elevated in the hypoxic CD8^+^ T cell cluster (Figure 5A). In contrast, other granzymes, including *GZMK* and *GZMM*, were significantly downregulated in the hypoxic CD8^+^ T cell cluster (Figure 5A). Additionally, *NKG7* and *CST7* (encoding Cystatin F), key regulators of cytolytic granule exocytosis and activity, were also reduced in the hypoxic cluster (Figure 5A). Together, these findings suggest that the overall cytolytic capacity of hypoxic CD8^+^ T cells might be impaired despite elevated *GZMB* expression.

In the context of chronic antigen exposure, CD8^+^ T cells undergo a progressive loss of effector function, transitioning from “progenitor exhausted” to “terminally exhausted” states [34,35,36]. We observed that the hypoxic CD8^+^ T cell cluster exhibited reduced expression of progenitor-associated genes, including *TCF7* (encoding TCF1), *EOMES*, *KLF2*, *LIMD2*, and *CD27*, compared with the normoxic CD8^+^ T cell cluster (Figure 5B). Conversely, the hypoxic CD8^+^ T cell cluster showed increased expression of genes associated with terminal exhaustion, such as *PRDM1* (encoding BLIMP1), *FKBP5*, *KLRB1*, *ITGA1*, and *ID2* (Figure 5C). Notably, expression of co-inhibitory receptors, including *PDCD1* (encoding PD-1), *CTLA4*, *HAVCR2* (encoding TIM-3), and *LAG3*, did not differ significantly between the two groups, though *TIGIT* expression was higher in the normoxic CD8^+^ T cell cluster (Figure 5D). Overall, these findings suggest that hypoxia is associated with a terminally differentiated, dysfunctional phenotype in CD8^+^ T cells.

## 4. Discussion

PDAC remains largely refractory to immunotherapies, in part due to its immunosuppressive TME and poor tumor antigenicity [1,2,3]. Hallmark features of PDAC include a dense fibroinflammatory stroma and profound hypoxia [1,12]. Consistent with previous reports [17,18,19], we find that intrinsic hypoxic exposure reduces the proliferative capacity of CD8^+^ T cells and increases the expression of co-inhibitory receptors. Importantly, when CD8^+^ T cells are exposed to hypoxia in the presence of cancer cell/CAF-derived factors, hypoxia cooperates with these extrinsic signals to further impair T cell fitness, as evidenced by increased cell death and reduced proliferation. In line with these findings, our analysis of human PDAC scRNA-seq data indicates that hypoxia is positively associated with apoptosis-related pathways and negative regulators of proliferation.

Hypoxia has been shown to enhance T cell function in certain contexts [17,19]. Consistent with this, we observed that hypoxic exposure increases IFNγ and GZMB expression in CD8^+^ T cells in the presence of cancer cell/CAF-secreted factors. However, the absolute number of effector T cells is markedly reduced under hypoxia due to impaired survival and proliferation. Furthermore, our analysis of human PDAC scRNA-seq data shows that the hypoxic CD8^+^ T cell cluster exhibits increased *GZMB* expression but decreased levels of *GZMK* and *GZMM*, as well as *NKG7*, a key regulator of cytotoxic granule exocytosis. Together, these data suggest that while hypoxia may enhance certain aspects of effector programming on a per-cell basis, it ultimately limits the overall pool of functional T cells within the TME.

A notable finding from our scRNA-seq analysis is that hypoxia correlates with reduced stemness and enhanced terminal differentiation in CD8^+^ T cells in PDAC. Additionally, consistent with recent studies implicating proteotoxic stress in T cell dysfunction [37,38], we observed a correlation between hypoxia and unfolded protein response signatures in CD8^+^ T cells. These findings raise the possibility that hypoxia promotes CD8^+^ T cell differentiation toward dysfunctional states. Notably, mitochondrial dysfunction has been shown to drive terminal T cell differentiation via glycolytic reprogramming mediated by HIF1α, a principal regulator of hypoxic adaptation [39]. Furthermore, BLIMP1 (encoded by *PRDM1*), a key transcriptional regulator of terminal exhaustion, has been shown to increase mitochondrial reactive oxygen species levels, thereby accelerating terminal differentiation [18]. Consistent with this, our scRNA-seq analysis revealed that hypoxic CD8^+^ T cells are enriched for the glycolysis signature and exhibit increased *PRDM1* expression, suggesting that hypoxia may promote terminal T cell exhaustion in PDAC through mechanisms involving mitochondrial dysfunction and metabolic reprogramming.

Hypoxia, in combination with tumor/CAF-derived factors, increased PD-1, TIM-3, and LAG-3 expression in vitro. However, expression of these co-inhibitory receptors was not significantly associated with the hypoxic CD8^+^ T cell cluster in the human PDAC dataset. This discrepancy may reflect limitations of our in vitro system, which relies on acute anti-CD3/CD28 stimulation and does not model chronic antigen exposure required for canonical exhaustion. Alternatively, terminally differentiated CD8^+^ T cells in hypoxic tumor regions may be short-lived and, therefore, underrepresented in steady-state scRNA-seq datasets.

We further examined whether tumor/CAF-derived factors produced under normoxic versus hypoxic conditions differentially regulate CD8^+^ T cell responses. While secreted factors from normoxic and hypoxic cancer cell–CAF co-cultures had comparable effects on T cell proliferation, survival, and co-inhibitory receptor expression, the factors from hypoxic co-cultures more strongly suppressed IFNγ expression, regardless of T cell oxygen conditions. These findings suggest that hypoxia reprograms tumor cells and CAFs to further suppress T cell effector function. Future studies will be needed to identify the specific proteins and/or metabolites induced under hypoxic conditions that mediate these effects. One candidate is IL-6, which is induced by hypoxia in pancreatic CAFs [23,27] and has been shown to inhibit cytotoxic effector differentiation of CD8^+^ T cells [40]. Ultimately, in vivo functional studies will be essential to determine how the spatial heterogeneity of the stroma and hypoxia in PDAC collectively regulate T cell fitness and differentiation.

## 5. Conclusions

Taken together, our data demonstrate that hypoxia not only exerts direct, cell-intrinsic effects on CD8^+^ T cells but also indirectly modulates them through extrinsic signals from tumor cells and CAFs. Our findings highlight the importance of considering both intrinsic hypoxic responses and extrinsic, context-dependent cues from the TME—including those dynamically altered by hypoxia—when designing effective immunotherapeutic strategies for PDAC.

## Data Availability

All data needed to evaluate the conclusions in the paper are present in the paper. This study did not generate new materials.

## Supplementary Materials

The following supporting information can be downloaded at: https://www.mdpi.com/article/10.3390/cancers18101508/s1, Figure S1. Absolute numbers of IFNγ^+^ cells are reduced in hypoxic CD8^+^ T cell cultures. Absolute numbers of live IFNγ^+^CD8^+^ T cells are shown for the same dataset presented as percentages in Figure 2B. Each dot represents an independent experiment. Data are mean ± SEM. *p* values were determined by two-way ANOVA. ** *p* < 0.01; *** *p* < 0.001.
