# Supplementary material for: Hypoxia Impairs CD8+ T Cell Fitness and Is Associated with a Dysfunctional CD8+ T Cell State in Pancreatic Cancer
Source: Cancers (Basel). 2026 May 8;18(10):1508. doi: 10.3390/cancers18101508 (PMC13204382; doi:10.3390/cancers18101508)
Supplement: Supplementary file 1 [file cancers-18-01508-s001.zip › cancers-4270851-Supplementary Materials.pdf]

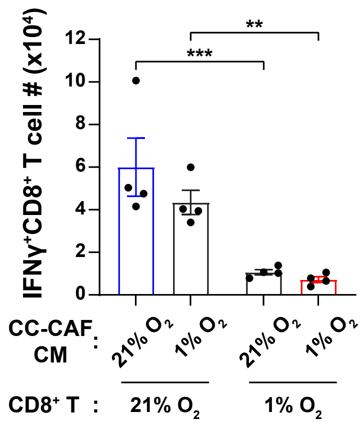

Figure S1. Absolute numbers of IFNγ⁺ cells are reduced in hypoxic CD8⁺ T cell cultures. Absolute numbers of live IFNγ⁺CD8⁺ T cells are shown for the same dataset presented as percentages in Figure 2B. Each dot represents an independent experiment. Data are mean ± SEM. *P* values were determined by two-way ANOVA. **$p < 0.01$; ***$p < 0.001$.
